# The Cost of Disease

**Published:** 1916-05

**Authors:** 


### EDITORIAL DEPARTMENT

MRS. F. E. DANIEL, Publisher and Managing Editor.

### The Cost of Disease.

Physicians everywhere are calling the attention of the public to the high cost of disease. Statistics show that the annual loss of labor and cost of medical attention in this country is more than a billion dollars more than it would be if proper methods were taken to prevent unnecessary diseases. Added to this it is estimated that a half billion dollars is spent for medicine taken without any prescription or advice from physicians, most of this money being worse than wasted. As an economic proposition, this great waste should be avoided. The only way to bring this about is by a systematic educational campaign under national and State direction. Such a campaign should have the active co-operation of every physician.

There is an erroneous belief extant that physicians do not want the people to be well, do not care to do anything to decrease preventable diseases. There may be a few quacks who hold to this narrow view, but all reputable, educated physicians know that the healthier the country becomes the better it will be for them. There will always be enough practice for physicians of ability, and such men had rather practice their profession in a healthy community, where disease is reduced to the minimum, and consequently where their patients are able to pay for their services. Therefore, the best physicians everywhere are cordially helping the State health officers in their efforts to rid the country of wasteful disease. The time will come when the country whose people have diseases that can be prevented by proper effort will be held in contempt if it does not do its duty in stopping the ravages of needless diseases. Wonderful progress in opinion and in practice has been made in this respect in the past few decades.

